# Is there a link between health care utilisation and subjective well-being? An exploratory study among older Danes

**DOI:** 10.1007/s10198-024-01691-1

**Published:** 2024-05-08

**Authors:** Maiken Skovrider Aaskoven, Trine Kjær, Dorte Gyrd-Hansen

**Affiliations:** DaCHE – Danish Centre for Health Economics, Department of Public Health, University of Southern Denmark, Campusvej 55, Odense M, 5230 Denmark

**Keywords:** Health care utilisation, Health-related quality of life, Subjective well-being, Ageing, Denmark, I1, I31

## Abstract

While extensive research has explored the influence of traditional factors such as socioeconomic position on health care utilisation, the independent role of an individual’s well-being in their health care seeking behaviour remains largely uncharted territory. In this study, we delve into the role of subjective well-being (SWB) in health care utilisation. We use a unique link between survey data from a representative group of Danish citizens aged 50-80 and administrative register data containing information on health care utilisation and sociodemographics. We explore whether SWB is a predictor of health care utilisation (general practice services) over and above health (as measured by health-related quality of life (HRQoL)). We find that the association between SWB and number of services provided in general practice differs across levels of HRQoL. Among those with lower HRQoL, we find a positive association between health care utilisation and SWB. Results hold true even when controlling for previous health care utilisation, suggesting that the mechanism is not driven by reverse causality. Our findings suggest that, in particular for vulnerable individuals in poor health and with poor SWB, the propensity to seek care is inappropriately low, and there is a need for more proactive supply-driven health care.

**Supplementary Information:**

The online version contains supplementary material available at 10.1007/s10198-024-01691-1.

## Highlights

SWB is a strong predictor of health care utilisation.

Individuals with low SWB seeks health care less often than those with high SWB.

Low SWB is a barrier to accessing health care for individuals with low HRQoL.

For individuals with perfect HRQoL, SWB acts as a need indicator.

The results are robust to controlling for previous health care utilisation.

**Supplementary Information:**

The online version contains supplementary material available at 10.1007/s10198-024-01691-1.

## Introduction

With an aging population in need of medical attention, it is becoming increasingly important to understand which factors determine health care utilisation if policy makers are to allocate health care resources equitably and cost-effectively. Demand for health care may be driven by factors that are not directly related to need for care. Financial, organisational, social and cultural barriers may limit the utilisation of services [1] and hinder one of the primary goals of health care systems: provision of health care according to need. Need is often proxied by individuals’ health status under the assumption that there is a strong correlation between health status and capacity to benefit from health care. This implies that individuals with poorer health are assumed to have a higher need for health care compared to individuals with better health [2, 3]^1^. In more recent years, subjective well-being (SWB) has been promoted as a more relevant measure of health care need than health-related quality of life (HRQoL). The primary argument being that it is a broader concept, which more precisely reflects individual overall utility [5]. HRQoL is most often based on individuals’ descriptions of their health state in terms of pain, depression, and ability to perform daily tasks and social activities. The (weighed) sum of the individuals’ performance on these health state dimensions produces a HRQoL score. In contrast, SWB poses questions about *satisfaction* with life overall or in relation to specific dimensions of life. HRQoL, when based on a pre-specified descriptive system, provides a more objective report, whereas SWB represents the individuals’ subjective assessment of their life situation broadly. The latter holistic view may allow for a more comprehensive assessment of an individual’s health care needs.

There is a large body of literature that shows that SWB is related to health outcomes. Lower levels of SWB have been linked to poorer physical health, early mortality, diabetes and cardiovascular diseases [6–8]. However, SWB has also been found to be associated with behavioural patterns relating to how individuals deal with health. Some studies have found that low SWB is associated with poorer coping behaviour [9, 10] and thus a lower ability to seek care when needed. Others have found that individuals with high SWB may demand less outpatient care as they are able to maintain healthy by other means, e.g., increased physical activity [11]. To the extent that SWB is not merely a more advanced measure of need for health care, but also signifies coping abilities and thus a potential personal barrier to optimal health care utilisation, the role of SWB is far more complex than anticipated. Low SWB may potentially entail a decreased ability (due to restrictions on abilities and resources) to seek health care when needed.

The evidence of SWB’s role in health care seeking behaviour is limited, but studies agree that SWB is negatively associated with health care utilisation. Harrison et al. [12] and Sears et al. [13] find that individuals with higher SWB have fewer hospital admissions, emergency room visits, and lower health care costs. Likewise, Straszewski et al. [14] show that inpatient hospital days, hospital admissions, outpatient visits, and emergency room visits decrease with increasing SWB, although their estimated associations are weaker than in other studies. Sidney et al. [15] find that minor improvements in SWB are associated with small savings in health care costs, and Riley et al. [16] observe that higher SWB at the county level reduces the county’s Medicare spending. While most studies focus on the secondary sector, few have investigated the relationship between SWB and health care utilisation in the primary sector. Al-Windi et al. [17] show that individuals with lower SWB have more appointments with a physician, while Kim et al. [18] show that an increase in life satisfaction reduces the number of doctor visits (including emergency room and clinic visits) significantly.

According to Grossman [19] individuals invest in their health to offset the deterioration of health over time, hence larger investments will lead to lower deterioration in health. One of the central elements of the Grossman model is that education enables individuals to produce health more efficiently. Likewise, SWB may represent a barrier to accessing health, and thus impact on individuals’ ability to invest in health. Low SWB may potentially impact on individuals’ propensity to invest in two ways. Investments may be too costly (the energy and effort required to seek care is not readily available). At the same, the propensity to invest in health may be low because the marginal benefits associated with health improvements are decreasing in SWB.

In order to understand the behavioural role of SWB, we investigate whether SWB is predominantly a separate need indicator in which case we would expect to observe a negative association between SWB and health care utilisation. Alternatively, SWB may mainly represent a barrier to investing in health due to poorer coping abilities or due to lesser returns of investment, in which case the association should be positive. Both mechanisms may be at play and vary depending on the level of HRQoL.

In this study, we exploit a unique link between a representative survey of older Danes and rich administrative register data. Our purpose is to assess whether and how SWB is a predictor of health care utilisation over and above health (as measured by HRQoL). Our dependent variable of choice is number of services provided in general practice (GP), as such services are more demand-driven (and thus more likely to be driven by patient circumstances) than the more specialised services. We also contribute to the literature by exploring whether there is a heterogenous relationship between SWB and health care utilisation across HRQoL levels. Moreover, we investigate the behavioural role of SWB on health care utilisation in a country with universal health coverage.

## Methods

In this study, we combine the versatility of the survey and the comprehensiveness of the Danish registers. Data is obtained from a cross-sectional survey conducted in spring 2019 on a representative group of Danish citizens aged 50-80. This data is supplemented with individual-level data from the administrative registers for the period 2016-2020.

### Data

Survey data was collected in spring 2019 (weeks 12-16) through a web-based questionnaire administered by Statistics Denmark using the digital mailbox for official communication from governmental agencies. We invited 15,072 individuals to participate, of which 6,807 individuals (45%) returned a fully completed questionnaire. An additional 323 individuals were excluded due to missing information, death or emigration during the study period (2016-2020), see Appendix Table A1 for further details. Thus, our final sample consists of 6,484 individuals. Appendix Table A2 shows that 47.3% are men, the average age is 64.5 years, 4.6% have a non-ethnic Danish background, 23.2% lives alone, the average number of children per individual is 1.8, the average number of years of education is 14.1, the average family income is DKK 337,426, and they have a mean Charlson Comorbidity Index of 0.43.

HRQoL and SWB are derived from the survey data. HRQoL is measured using the descriptive system EQ-5D-5L with Danish weights [20, 21]. SWB is evaluated by the general life satisfaction question ‘How satisfied are you with your life as a whole?’. A more detailed description of the key variables can be found in Table 1. Data on health care utilisation, age, gender, ethnicity, living arrangement, children, municipality, education and income are obtained from the registers. Health care utilisation is measured as number of services provided in general practice in the weeks 17-52 in 2019. The other covariates are measured in 2019, the same year as we measure HRQoL and SWB. Please refer to Appendix Table A3 for additional details on these variables.

**Table 1:** Definitions of variables

Variable	Where	Definition	Year
HRQoL	Survey	Measured using EQ-5D-5L [20] designed to assess HRQoL across five dimensions: mobility, self-care, usual activities, pain/discomfort, and anxiety/depression, with five levels of severity (from no problems to extreme problems) for each dimension. EQ-5D-5L incorporates preference-based weights assigned to different health states by the general population in the sum score, with anchor points of 0 for death and 1 for perfect health. Danish weights are applied [21].	2019
SWB	Survey	Overall SWB is measured using the life satisfaction question ‘How satisfied are you with your life as a whole?’ on a 11-point end-defined response scale, with numerical ratings ranging from 0 (extremely dissatisfied) to 10 (extremely satisfied). SWB scores are converted into a 0-100 point scale.	2019
Health care utilisation	Register (SSSY)	Number of services provided in general practice. All types of services are included. Only normal office hours are included (Monday to Friday, 8AM to 16PM). Health care utilisation is measured on a weekly basis, and only weeks following the completion of the survey collection are included (weeks 17-52).	2019 and 2020 (robustness check)
Previous health care utilisation	Register (SSSY)	Average number of services provided in general practice over the three previous years.	2016-2018 and 2017-2019 (robustness check)

Note: The table provides the definitions of the key variables used in the study

### Analyses

Our main specification involves regressing SWB on health care utilisation using ordinary least squares with robust standard errors. We run two specifications: (1) we enter SWB as an independent covariate, and (2) we allow the effect of SWB to vary with HRQoL by adding an interaction term between HRQoL and SWB. For both specifications, we introduce our covariates in a stepwise manner. First, we introduce SWB alongside the traditional need indicator, HRQoL. Subsequently, we introduce other sociodemographic covariates that may act as confounders (age, gender, ethnicity, living arrangement, children, municipality, education, and family income). Finally, we control for prior health care utilisation (average GP services in 2016-2018). While it is likely that we overcontrol in these regressions, the results are indicative of a causal pathway.

We perform five robustness checks (see Appendix Table A5-A8). First, we exclude individuals who did not receive any services provided in GP to verify that the results are not driven by these outliers. Second, we exclude individuals with perfect health (i.e., HRQoL equal to 1) to confirm that the results are not driven by these individuals. Third, we weigh our regressions to recreate representativeness (se details in Appendix Table A3). Fourth, we add survey completion time as a covariate to ensure that our results are not driven by less robust responses. Finally, we conduct the analysis on 2020 utilisation data during a period affected by the COVID-19 pandemic (weeks 17-52) to verify the stability of SWB as a predictor of health care utilisation.

## Results

Health care utilisation per individual during weeks 17-52 in 2019 ranges from 0 (13.8%) to 169 GP services, with a mean of 8.69 services (SD = 9.51). Average HRQoL is 0.88, ranging from -0.63 to 1 (SD = 0.18). SWB ranges from 0 to 100, with an average of 82.0 (SD = 17.06). Further details are provided in Appendix Table A4. Appendix Fig. A1 and Fig. A2 show variation in health care utilisation across HRQoL and level of SWB, respectively. The correlation between HRQoL and SWB is 0.49 (Appendix Fig. A3).

**Fig. 1:**
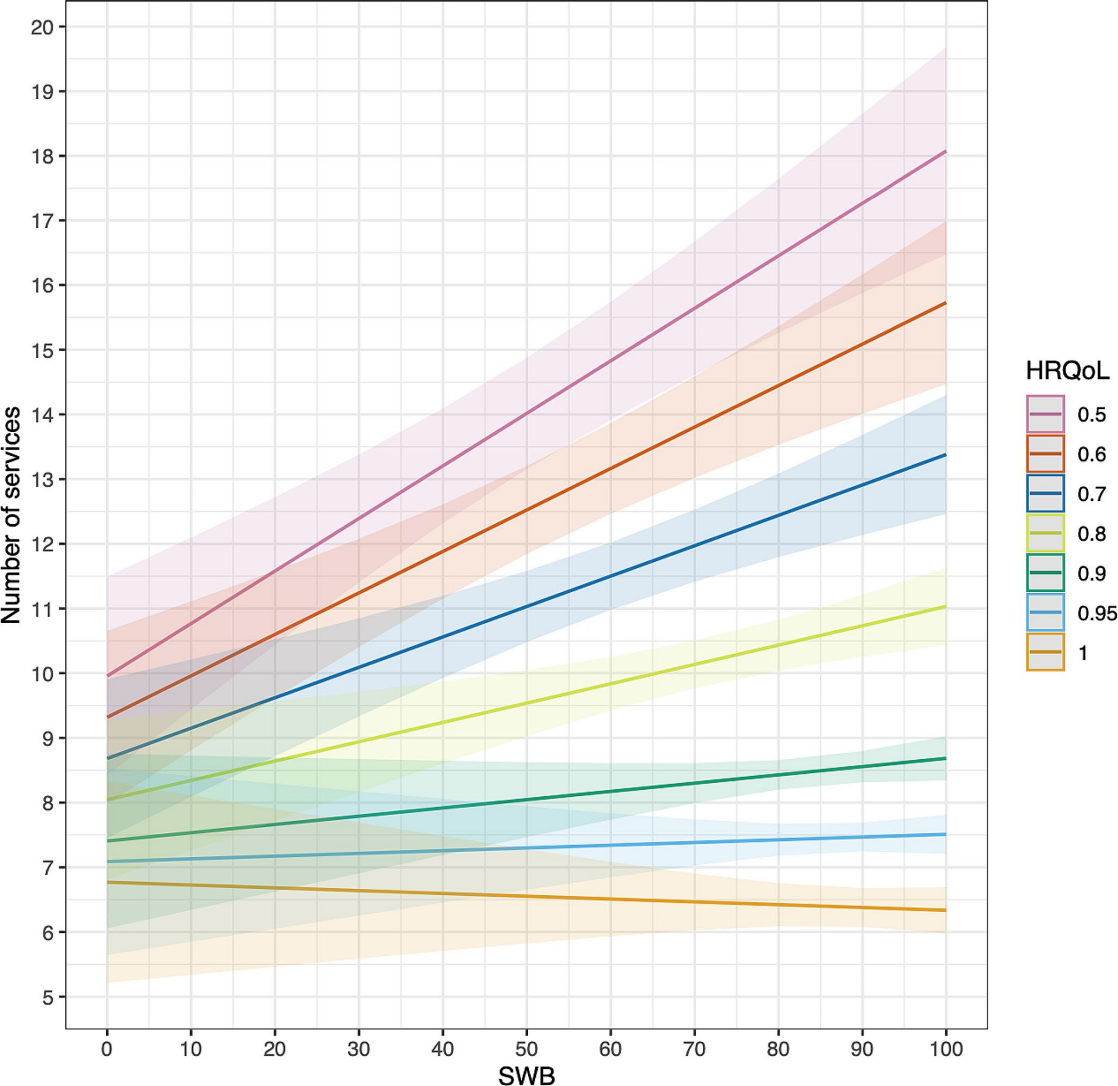
Predicted change in health care utilisation as a function of SWB and HRQoL. Note: The figure displays predicted values of health care utilisation as a function of SWB and HRQoL along with their 95% confidence intervals (robust). We do not control for other covariates in the model (corresponding to column (2) in Table 2)

Results show that SWB is a strong predictor of health care utilisation over and above classic predictors such as HRQoL and age. As expected, HRQoL is negatively associated with health care utilisation, whereas age is positively associated with it (Table 2). A key finding is that, on average, SWB tends to have a positive association with health care utilisation. This implies that individuals with low SWB (all else equal) seek health care less often than those with high SWB. When allowing for heterogenous effects of SWB across HRQoL, we find that the positive association is strongest for individuals with low levels of HRQoL. This suggests that low SWB is a stronger barrier to accessing health care for these individuals (for an illustration, see predictions in Fig. 1). Importantly, the observed patterns are robust to controlling for previous health care utilisation, implying that the relationship may be causal. Our results are robust to other specifications (see Appendix Table A5-A8). Interestingly, we observe the same associations between SWB and health care utilisation in 2020 (see Appendix Table A9).

**Table 2:** Marginal change in health care utilisation

	Dependent variable: Number of services provided in GP					
	(1)	(2)	(3)	(4)	(5)	(6)
Intercept	21.2043***	13.1421***	6.0359***	-2.3935	0.0146	-4.2616**
	(0.9909)	(1.5795)	(1.8747)	(2.2976)	(1.3482)	(1.7626)
HRQoL	-17.2810***	-6.3725***	-16.3515***	-5.1754***	-5.6694***	-0.0173
	(1.2880)	(1.9472)	(1.2400)	(1.9239)	(0.9310)	(1.5762)
SWB	0.0318***	0.1668***	0.0123	0.1503***	0.0167**	0.0874***
	(0.0085)	(0.0261)	(0.0085)	(0.0254)	(0.0067)	(0.0198)
HRQoL * SWB		-0.1711***		-0.1753***		-0.0897***
		(0.0282)		(0.0276)		(0.0218)
Male			-0.7418***	-0.7524***	0.1478	0.1367
			(0.2160)	(0.2155)	(0.1785)	(0.1779)
Age			0.2498***	0.2512***	0.0757***	0.0775***
			(0.0144)	(0.0144)	(0.0118)	(0.0118)
Non-ethnic Danish			-1.2215***	-1.0062**	-0.5639*	-0.4579
			(0.4241)	(0.4130)	(0.3385)	(0.3381)
Lives alone			0.6824**	0.6852**	0.4449*	0.4479*
			(0.3010)	(0.2997)	(0.2375)	(0.2371)
Children			0.0491	0.0447	-0.0284	-0.0302
			(0.1098)	(0.1088)	(0.0860)	(0.0855)
Education			-0.0953**	-0.0937**	-0.0008	-0.0006
			(0.0469)	(0.0466)	(0.0382)	(0.0381)
Family income			-0.0007***	-0.0006***	-0.0001	-0.0001
			(0.0002)	(0.0002)	(0.0002)	(0.0002)
Control for municipality			✓	✓	✓	✓
Control for previous health care utilisation					✓	✓
No. of observations	6,484	6,484	6,484	6,484	6,484	6,484

Note: The table shows regression coefficients and their robust standard errors in parentheses. * 10%, ** 5% and *** 1% significance level

## Discussion

Our results demonstrate that SWB plays a role in health care seeking behaviour, and that there are oppositely directed mechanisms at play. For individuals with perfect HRQoL, a higher SWB tends to be associated with lower health care utilisation. This suggests that SWB is likely to be an indicator of self-perceived need, implying that these individuals to some degree appear to prioritise their use of health care services appropriately. However, for individuals with poorer health, the picture is very different. For these individuals, a higher SWB is associated with increased use of health care services, suggesting that when health is poor and well-being is low, a relative higher level of SWB is required to enable access to health care, and thus investment in health. This finding suggests that for vulnerable individuals in poor health and with poor SWB, the propensity to seek care is likely to be inappropriately low, and there is a need for more proactive supply-driven health care.

To verify the robustness of our results, we replicate the analysis using health care utilisation data from 2020 and find very similar associations. These results demonstrate that even under unusual circumstances (COVID-19), where demand for GP services may be impacted (e.g., due to fear of infection, increased need for reassurance, or reluctance to use GP services out of consideration for others), SWB continues to play the same role. Therefore, our results suggest that SWB is likely to be barrier to accessing health care in many different contexts. It should be noted that our findings are based on a sample of 50-80-year-olds of which 73.1% are in non-perfect health. We cannot exclude the possibility that the role of SWB may differ for younger age groups.

Our results are not aligned with those reported in other studies. We find that SWB is a strong predictor of health care utilisation and that there, on average, is a positive association between SWB and use of services provided in GP. This may partly be due to our choice of covariates and dependent variable. In contrast to other studies, we control for HRQoL and thus go one step further in estimating SWB as an individual predictor of health care utilisation beyond health need. Moreover, other studies have focused on health care utilisation in the secondary sector [e.g., 12, 14], and/or have used health care costs as their dependent variable [e.g., 15, 16]. These outcomes are likely less patient-driven, and may not accurately reflect individuals’ demand for health care. The discrepancy with prior literature may also be attributed to the institutional setting. The majority of prior studies are based on US data, where there is normally some degree of co-payment for GP visits. In Denmark, where GP services are free of charge and income does not constitute a barrier, the impact of other more personal barriers, such as low SWB, may become more pronounced.

We cannot rule out the possibility that SWB might mediate other personal barriers, e.g., personality traits, or that SWB might be confounded by other structural barriers, e.g., employment or caregiving demands, or transportation needs. Such mechanisms may be at play even after we have controlled for a range of confounders, but this does not undermine our finding that SWB is an independent predictor of heath care utilisation.

### Limitations and scope for future research

The use of administrative data is a strength as health care utilisation is not self-reported, and we can take advantage of the longitudinal nature of the data. However, the survey is cross-sectional, meaning that we only observe SWB at a single point in time. This makes it challenging to draw causal interpretations, as the causal pathways cannot be uniquely identified. To control for the possibility that lower health care utilisation might lead to lower SWB, we run a specification where we control for health care utilisation predating SWB measurement. This specification yields similar results. While it is likely that we overcontrol in these regressions, the results support the notion that it is SWB that affects health care utilisation, and not vice versa.

It should be noted that we are only able to estimate the net effect of the two likely opposing behavioural mechanisms (need vs. barrier). Therefore, when we observe a positive association between SWB and health care utilisation, it merely demonstrates that the barrier mechanism plays the dominant role. We cannot exclude that our analyses may be subject to selection bias. It is likely that our pool of respondents has a higher HRQoL, higher SWB, and may also (all else equal) have a greater propensity to seek health care than the Danish population of 50-80-year-olds. Despite this potential selection bias, we are able to identify a link between SWB and health care utilisation, albeit it may be underestimated. Furthermore, the association between health care utilisation and SWB may be subject to confounding. We seek to overcome this by including a range of sociodemographic characteristics in our analyses. While multicollinearity could be a concern, there is only a low to moderate correlation between the covariates (see Appendix Fig. A4). We also calculated variance inflation factors, which did not detect multicollinearity (results not shown).

In conclusion, we believe we have, with a high degree of plausibility, identified a potential adverse effect of experiencing low SWB, which calls for further exploration. More research, particularly research providing stronger causal evidence, is warranted to understand the role that SWB plays in relation to demand-driven health care utilisation.

## Electronic supplementary material

Below is the link to the electronic supplementary material.

Supplementary Material 1

## Data Availability

The datasets generated and/or analysed during the current study are not publicly available due to Danish Data Protection Legislation. Only Danish research environments can be granted authorisation to Danish administrative registers.

## Footnotes

1 The definition of health care need has been discussed to a great extend in the health economic literature. There are two main definitions: individuals’ initial health state or individuals’ capacity to benefit [2–4].
