# Supplementary material for: Is there a link between health care utilisation and subjective well-being? An exploratory study among older Danes
Source: Eur J Health Econ. 2024 May 8;26(1):87–93. doi: 10.1007/s10198-024-01691-1 (PMC11743418; doi:10.1007/s10198-024-01691-1)
Supplement: Supplementary file 1 — Supplementary Material 1 [file 10198_2024_1691_MOESM1_ESM.pdf]

Is there a link between health care utilisation and subjective well-being? An exploratory study among older Danes

Maiken Skovrider AASKOVEN[1,*], Trine KJÆR[1], Dorte GYRD-HANSEN[1]
[1] DaChE – Danish Centre for Health Economics, Department of Public Health, University of Southern Denmark, Campusvej 55, 5230 Odense M, Denmark
[*] Corresponding author: maaskoven@health.sdu.dk

Supplementary material: Appendix

Table A1 Exclusions of study participants from the study population

Exclusion criterion	Count
Not living in Denmark in 2017-2018	34
Dead or emigrated after survey	89
Missing information on SWB	3
Missing information on HRQoL	3
Missing information on education	51
Missing information on family income	143
Total	323

Note: The table shows the exclusion criteria and number of participants dropped from the analyses.

Table A2 Means of covariates and health information for the study population

	Study population
N	6,484
Male (n (%))	3,069 (47.33)
Age (mean (SD))	64.498 (8.157)
Non-ethnic Danish (n (%))	296 (4.57)
Lives alone (n (%))	1,506 (23.23)
Number of children (mean (SD))	1.826 (1.041)
Peripheral Denmark (n (%))	1,282 (19.77)
Length of education in years (mean (SD))	14.098 (2.720)
Family income in 1000 DKK (mean (SD))	337.426 (348.841)
SWB (mean (SD))	82.030 (17.059)
HRQoL (mean (SD))	0.875 (0.177)
Charlson Comorbidity Index (mean (SD))	0.428 (1.066)
Services provided by GP in weeks 17-52 in 2019 (mean (SD))	8.690 (9.509)
Average services provided by GP in 2016-2018 (mean (SD))	11.546 (11.073)

Note: The table shows the means and proportions of the covariates and selected health information for the study population.

Table A3 Definition of variables

Variable	Where	Definition	Year
Health care utilisation	Register (SSSY)	Number of services provided in general practice. All types of services are included. Only normal office hours are included (Monday to Friday, 8AM to 16PM). Health care utilisation is measured on a weekly basis, and only weeks following the completion of the survey collection are included (weeks 17-52).	2019
Previous health care utilisation	Register (SSSY)	Average number of services provided in general practice over the three previous years.	2016-2018
Health care utilisation during COVID-19	Register (SSSY)	Number of services provided in weeks 17-52 in 2020. This is also after the first major lockdown due to COVID-19 (weeks).	2020
Previous health care utilisation during COVID-19	Register (SSSY)	Average number of services provided in general practice over the three previous years.	2017-2019
HRQoL	Survey	Measured using EQ-5D-5L [1] designed to assess HRQoL across five dimensions: mobility, self-care, usual activities, pain/discomfort, and anxiety/depression, with five levels of severity (from no problems to extreme problems) for each dimension. EQ-5D-5L incorporates preference-based weights assigned to different health states by the general population in the sum score, with anchor points of 0 for death and 1 for perfect health. Danish weights are applied [2].	2019
SWB	Survey	Overall SWB is measured using the life satisfaction question 'How satisfied are you with your life as a whole?' on a 11-point end-defined response scale, with numerical ratings ranging from 0 (extremely dissatisfied) to 10 (extremely satisfied). SWB scores are converted into a 0-100 point scale.	2019
Age	Register (BEF)	Participant's age.	2019
Gender	Register (BEF)	Participant's legal gender (male or female).	2019
Ethnicity	Register (BEF)	Participant's ethnicity is evaluated as either non-ethnic Danish or ethic Danish. Non-ethnic Danes include immigrants and descendants.	2019
Living arrangement	Register (BEF)	Whether the participant lives alone or not (includes both living with a partner or a child).	2019
Children	Register (BEF)	How many legal children the participant has (includes both adopted and biological children).	2019
Municipality	Register (BEF)	Which municipality the participant lives in.	2019
Peripheral Denmark	Register (BEF)	Whether the municipality that the participant lives in is defined as a peripheral municipality ('yderkommune') by the Danish tax authorities.	2019
Education	Register (UDDA)	Measured as highest achieved education length in months. Converted into years of education.	2019
Family income	Register (FAIK)	The equivalent disposable income of the participant's family in DKK (the variable corrects for the size of the family), converted into 1000 DKK.	2019

Table A3 *(Continued)*

Charlson Comorbidity Index	Register (LPR)	An index that quantifies the burden of comorbidities in participants by assigning weights to various chronic conditions, e.g., heart disease, diabetes, and cancer, assessing their severity and impact on mortality. A higher score indicates a greater overall health risk. It is constructed for a five-year period with weights from Quan et al. [3].	2014-2018
Survey completion time	Survey	Number of minutes the respondent used to complete the survey. Rounded to the nearest minute.	2019
Survey weights	Statistics Denmark	The survey weights are calculated by Statistics Denmark and are based on gender, age, family composition, education, socio-economic status, family income, type of immigrant, and labour market affiliation.	2019

Note: The table shows the definition of the variables used in the study.

Table A4 Descriptive statistics of selected variables

	Minimum	1st quartile	Median	Mean	3rd quartile	Maximum	Standard deviation
Number of services	0.00	2.00	6.00	8.69	12.00	169.00	9.51
HRQoL	-0.6300	0.8470	0.9280	0.8754	1.0000	1.0000	0.18
SWB	0.00	80.00	90.00	82.03	90.00	100.00	17.06

Note: This table shows minimum, maximum, quartiles, mean value, and standard deviations for health care utilisation in weeks 17-52 in 2019, HRQoL, and SWB.

Table A5 Marginal change in health care utilisation when conditioning on having at least one service provided

	Dependent variable: Number of services provided by GP					
	(1)	(2)	(3)	(4)	(5)	(6)
Intercept	20.9539***	13.4571***	7.6601***	-0.0739	1.4467	-2.7442
	(1.0115)	(1.5829)	(2.0032)	(2.4009)	(1.4804)	(1.8590)
HRQoL	-15.0557***	-4.7119**	-14.5488***	-4.0896**	-5.6075***	0.0563
	(1.3345)	(1.9748)	(1.2776)	(1.9462)	(0.9778)	(1.6053)
SWB	0.0264***	0.1527***	0.0119	0.1396***	0.0185**	0.0884***
	(0.0091)	(0.0265)	(0.0091)	(0.0257)	(0.0075)	(0.0203)
HRQoL * SWB		-0.1627***		-0.1645***		-0.0901***
		(0.0287)		(0.0281)		(0.0224)
Male			-0.4101*	-0.4192*	0.3153	0.3053
			(0.2366)	(0.2361)	(0.2027)	(0.2022)
Age			0.2156***	0.2170***	0.0645***	0.0664***
			(0.0158)	(0.0157)	(0.0131)	(0.0130)
Non-ethnic Danish			-1.1489**	-0.9357**	-0.6050	-0.4920
			(0.4586)	(0.4471)	(0.3882)	(0.3873)
Lives alone			0.8864***	0.8913***	0.5500**	0.5550**
			(0.3288)	(0.3274)	(0.2670)	(0.2665)
Children			-0.0358	-0.0379	-0.0683	-0.0693
			(0.1232)	(0.1223)	(0.1003)	(0.0998)
Education			-0.1244**	-0.1239**	-0.0204	-0.0208
			(0.0516)	(0.0514)	(0.0435)	(0.0434)
Family income			-0.0007***	-0.0007***	-0.0001	-0.0001
			(0.0002)	(0.0002)	(0.0002)	(0.0002)
Control for municipality			✓	✓	✓	✓
Control for previous health care utilisation					✓	✓
No. of observations	5,591	5,591	5,591	5,591	5,591	5,591

Note: The table shows regression coefficients and their robust standard errors in parentheses. * 10%, ** 5% and *** 1% significance level.

Table A6 Marginal change in health care utilisation when excluding individuals with perfect HRQoL

	Dependent variable: Number of services provided by GP					
	(1)	(2)	(3)	(4)	(5)	(6)
Intercept	20.3729***	13.0861***	3.2454	-3.9979	-1.2418	-5.5024***
	(1.0706)	(1.5997)	(2.1754)	(2.5778)	(1.5895)	(1.9653)
HRQoL	-16.2103***	-5.7515***	-15.1444***	-5.0173**	-5.4671***	0.4702
	(1.4313)	(2.0635)	(1.3758)	(2.0776)	(1.0326)	(1.6941)
SWB	0.0342***	0.1612***	0.0154	0.1386***	0.0182**	0.0911***
	(0.0095)	(0.0285)	(0.0095)	(0.0279)	(0.0076)	(0.0216)
HRQoL * SWB		-0.1700***		-0.1648***		-0.0975***
		(0.0323)		(0.0318)		(0.0249)
Male			-0.9272***	-0.9243***	0.1114	0.1071
			(0.2682)	(0.2678)	(0.2258)	(0.2250)
Age			0.2725***	0.2727***	0.0890***	0.0901***
			(0.0179)	(0.0178)	(0.0147)	(0.0147)
Non-ethnic Danish			-1.2698**	-1.0591**	-0.7230*	-0.6015
			(0.5086)	(0.4962)	(0.4092)	(0.4086)
Lives alone			0.7390**	0.7222**	0.5516*	0.5428*
			(0.3678)	(0.3662)	(0.2986)	(0.2982)
Children			0.0485	0.0458	0.0008	-0.0005
			(0.1378)	(0.1367)	(0.1098)	(0.1092)
Education			-0.1017*	-0.0962	0.0006	0.0033
			(0.0597)	(0.0594)	(0.0488)	(0.0487)
Family income			-0.0009***	-0.0008***	-0.0002	-0.0002
			(0.0003)	(0.0003)	(0.0002)	(0.0002)
Control for municipality			✓	✓	✓	✓
Control for previous health care utilisation					✓	✓
No. of observations	4,737	4,737	4,737	4,737	4,737	4,737

Note: The table shows regression coefficients and their robust standard errors in parentheses. * 10%, ** 5% and *** 1% significance level.

Table A7 Marginal change in health care utilisation when applying survey weights on the regressions

	Dependent variable: Number of services provided by GP					
	(1)	(2)	(3)	(4)	(5)	(6)
Intercept	20.5221***	13.0895***	4.2679**	-3.5715	-0.8164	-5.0876***
	(1.1409)	(1.5737)	(1.9732)	(2.3664)	(1.4072)	(1.8394)
HRQoL	-16.9840***	-6.4730***	-16.1917***	-5.5386***	-5.3294***	0.4454
	(1.6515)	(1.9983)	(1.5277)	(1.9671)	(1.0370)	(1.6667)
SWB	0.0374***	0.1701***	0.0167*	0.1507***	0.0179**	0.0915***
	(0.0101)	(0.0303)	(0.0098)	(0.0287)	(0.0078)	(0.0221)
HRQoL * SWB		-0.1725***		-0.1744***		-0.0959***
		(0.0320)		(0.0305)		(0.0242)
Male			-0.8399***	-0.8512***	0.0624	0.0494
			(0.2384)	(0.2381)	(0.1965)	(0.1950)
Age			0.2532***	0.2556***	0.0766***	0.0793***
			(0.0169)	(0.0168)	(0.0129)	(0.0128)
Non-ethnic Danish			-1.3493***	-1.0832**	-0.5249	-0.3847
			(0.4429)	(0.4290)	(0.3440)	(0.3445)
Lives alone			0.9267***	0.9263***	0.6055**	0.6077**
			(0.3351)	(0.3328)	(0.2542)	(0.2535)
Children			0.0150	0.0014	-0.0394	-0.0464
			(0.1210)	(0.1198)	(0.0939)	(0.0932)
Education			-0.0736	-0.0693	-0.0053	-0.0034
			(0.0522)	(0.0517)	(0.0412)	(0.0411)
Family income			-0.0007***	-0.0006**	0.0000	0.0000
			(0.0002)	(0.0002)	(0.0002)	(0.0002)
Control for municipality			✓	✓	✓	✓
Control for previous health care utilisation					✓	✓
No. of observations	6,484	6,484	6,484	6,484	6,484	6,484

Note: The table shows regression coefficients and their robust standard errors in parentheses. * 10%, ** 5% and *** 1% significance level.

Table A8 Marginal change in health care utilisation when including a variable for survey completion time

	Dependent variable: Number of services provided by GP					
	(1)	(2)	(3)	(4)	(5)	(6)
Intercept	19.5542***	11.7250***	5.8439***	-2.4987	-0.1470	-4.3640**
	(1.0680)	(1.6214)	(1.9051)	(2.3144)	(1.3761)	(1.7832)
HRQoL	-16.9303***	-6.2746***	-16.3107***	-5.1902***	-5.6357***	-0.0317
	(1.2931)	(1.9419)	(1.2476)	(1.9196)	(0.9361)	(1.5741)
SWB	0.0308***	0.1628***	0.0124	0.1499***	0.0168**	0.0869***
	(0.0085)	(0.0260)	(0.0085)	(0.0254)	(0.0067)	(0.0198)
HRQoL * SWB		-0.1673***		-0.1746***		-0.0891***
		(0.0281)		(0.0276)		(0.0218)
Male			-0.7309***	-0.7444***	0.1570	0.1445
			(0.2169)	(0.2164)	(0.1794)	(0.1788)
Age			0.2463***	0.2487***	0.0728***	0.0751***
			(0.0153)	(0.0153)	(0.0124)	(0.0124)
Non-ethnic Danish			-1.2854***	-1.0531**	-0.6178*	-0.5036
			(0.4257)	(0.4149)	(0.3422)	(0.3417)
Lives alone			0.6839**	0.6862**	0.4462*	0.4489*
			(0.3008)	(0.2996)	(0.2373)	(0.2370)
Children			0.0489	0.0446	-0.0286	-0.0303
			(0.1098)	(0.1088)	(0.0860)	(0.0855)
Education			-0.0912*	-0.0907*	0.0026	0.0023
			(0.0476)	(0.0474)	(0.0390)	(0.0389)
Family income			-0.0007***	-0.0006***	-0.0001	-0.0001
			(0.0002)	(0.0002)	(0.0002)	(0.0002)
Control for municipality			✓	✓	✓	✓
Control for previous health care utilisation					✓	✓
No. of observations	6,484	6,484	6,484	6,484	6,484	6,484

Note: The table shows regression coefficients and their robust standard errors in parentheses. * 10%, ** 5% and *** 1% significance level.

Table A9 Marginal change in health care utilisation when health care utilisation is measured in 2020

	Dependent variable: Number of services provided by GP					
	(1)	(2)	(3)	(4)	(5)	(6)
Intercept	23.0015***	13.2217***	6.4338***	-3.8313	0.5645	-4.2776*
	(0.9662)	(1.7938)	(2.2058)	(2.6864)	(1.7522)	(2.3116)
HRQoL	-17.7077***	-4.4753**	-16.6122***	-3.0024	-4.4468***	1.9381
	(1.2227)	(2.2747)	(1.1838)	(2.2221)	(0.8968)	(2.0656)
SWB	0.0267***	0.1903***	0.0003	0.1684***	0.0009	0.0809***
	(0.0098)	(0.0297)	(0.0098)	(0.0289)	(0.0078)	(0.0248)
HRQoL * SWB		-0.2076***		-0.2135***		-0.1017***
		(0.0333)		(0.0323)		(0.0283)
Male			-0.9863***	-0.9991***	-0.1531	-0.1658
			(0.2333)	(0.2327)	(0.1891)	(0.1890)
Age			0.3174***	0.3191***	0.1185***	0.1209***
			(0.0152)	(0.0152)	(0.0123)	(0.0123)
Non-ethnic Danish			-1.5522***	-1.2900***	-0.8303**	-0.7112**
			(0.4916)	(0.4775)	(0.3550)	(0.3543)
Lives alone			0.3377	0.3411	0.0471	0.0510
			(0.3044)	(0.3020)	(0.2308)	(0.2300)
Children			0.1493	0.1440	0.0660	0.0641
			(0.1177)	(0.1171)	(0.0896)	(0.0895)
Education			-0.0871*	-0.0852*	-0.0027	-0.0024
			(0.0458)	(0.0455)	(0.0351)	(0.0351)
Family income			-0.0009***	-0.0008***	-0.0003	-0.0003
			(0.0003)	(0.0003)	(0.0002)	(0.0002)
Control for municipality			✓	✓	✓	✓
Control for previous health care utilisation					✓	✓
No. of observations	6,484	6,484	6,484	6,484	6,484	6,484

Note: The table shows regression coefficients and their robust standard errors in parentheses. * 10%, ** 5% and *** 1% significance level.

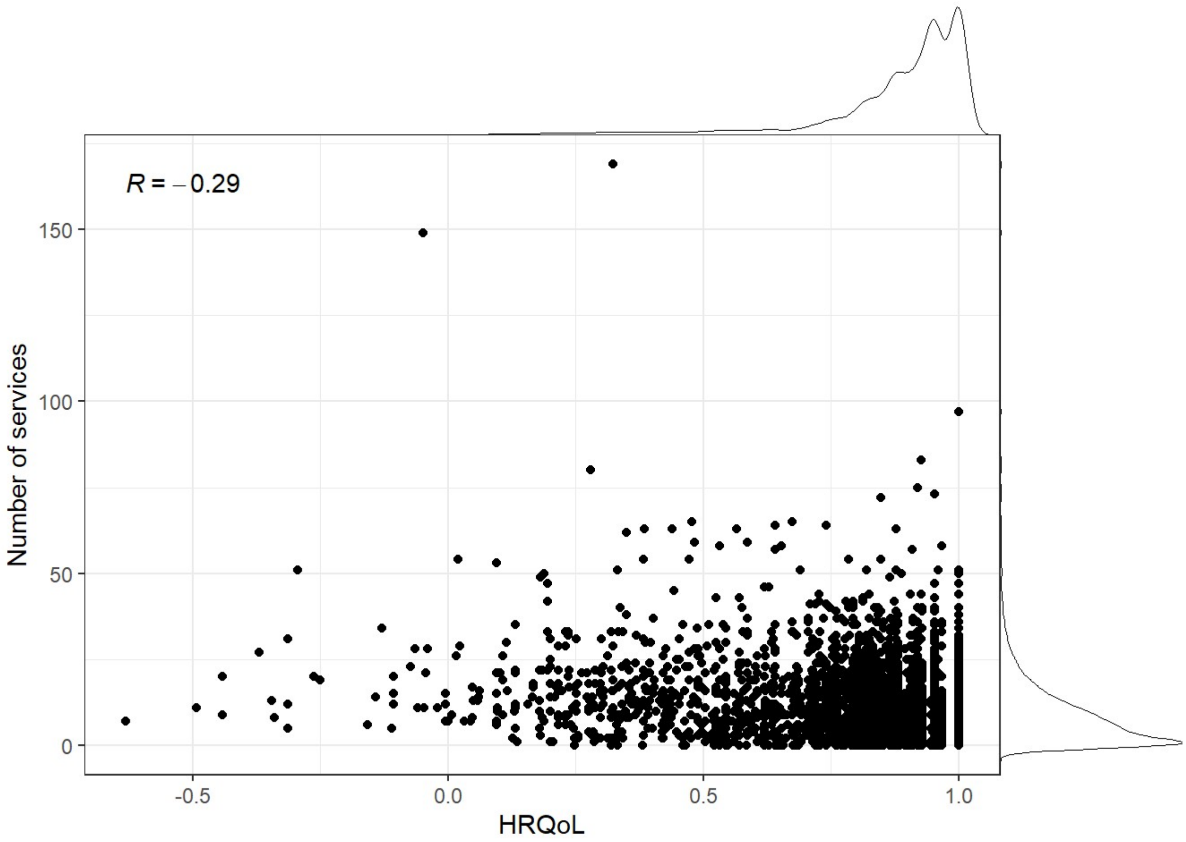

Fig. A1 Health care utilisation and HRQoL

Note: The figure displays a scatterplot of health care utilisation and HRQoL with their distributions on the sides of the scatterplot. Correlation displayed by R. 893 individuals (13.77%) do not receive any services provided by GP in the weeks 17-52 in 2019.

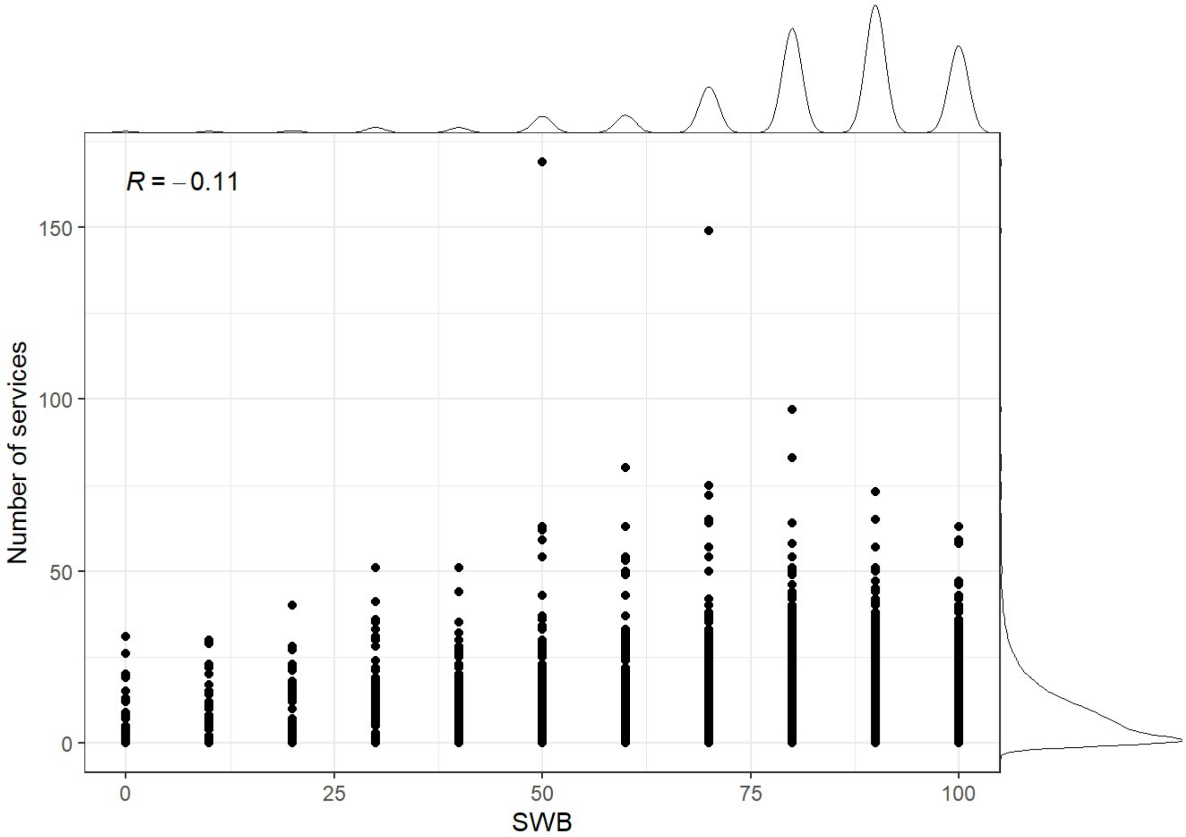

Fig. A2 Health care utilisation and SWB

Note: The figure displays a scatterplot of health care utilisation and SWB with their distributions on the sides of the scatterplot. Correlation displayed by R. 893 individuals (13.77%) do not receive any services provided by GP in the weeks 17-52 in 2019.

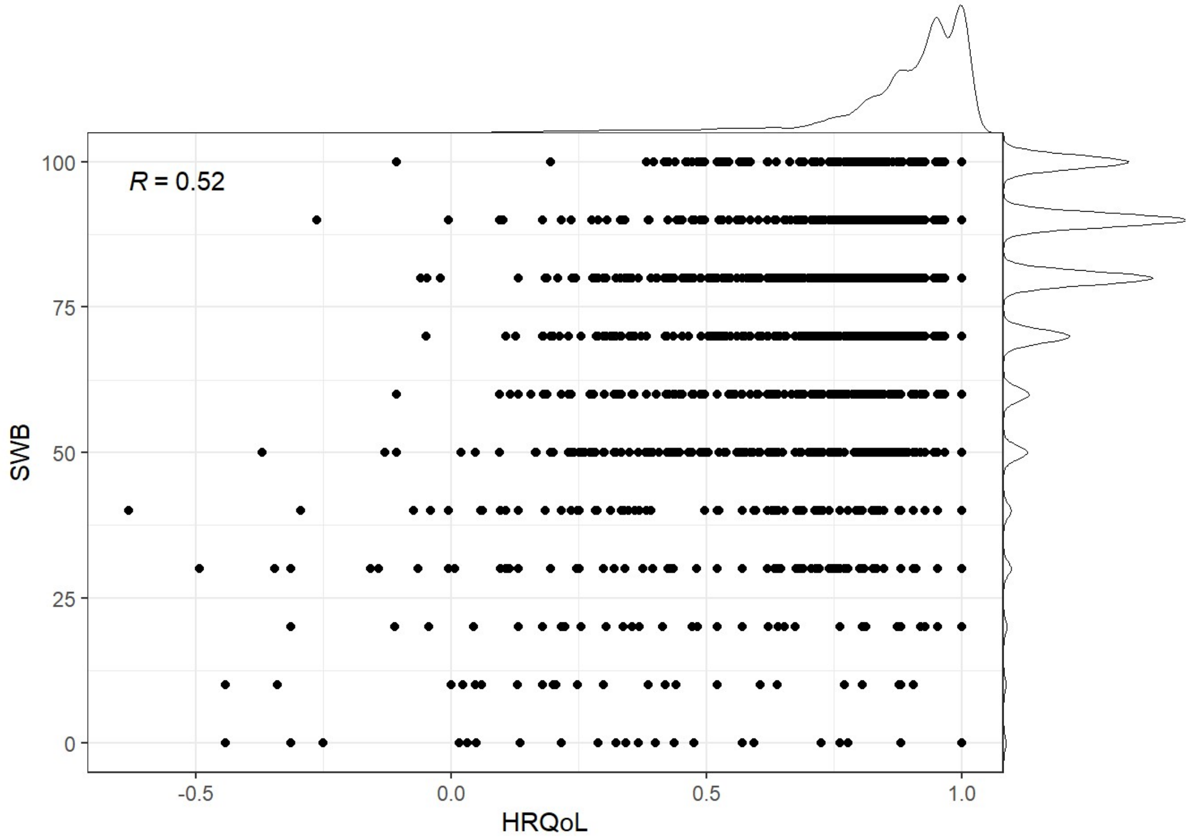

Fig. A3 SWB and HRQoL

Note: The figure displays a scatterplot of SWB and HRQoL with their distributions on the sides of the scatterplot. Correlation displayed by R.

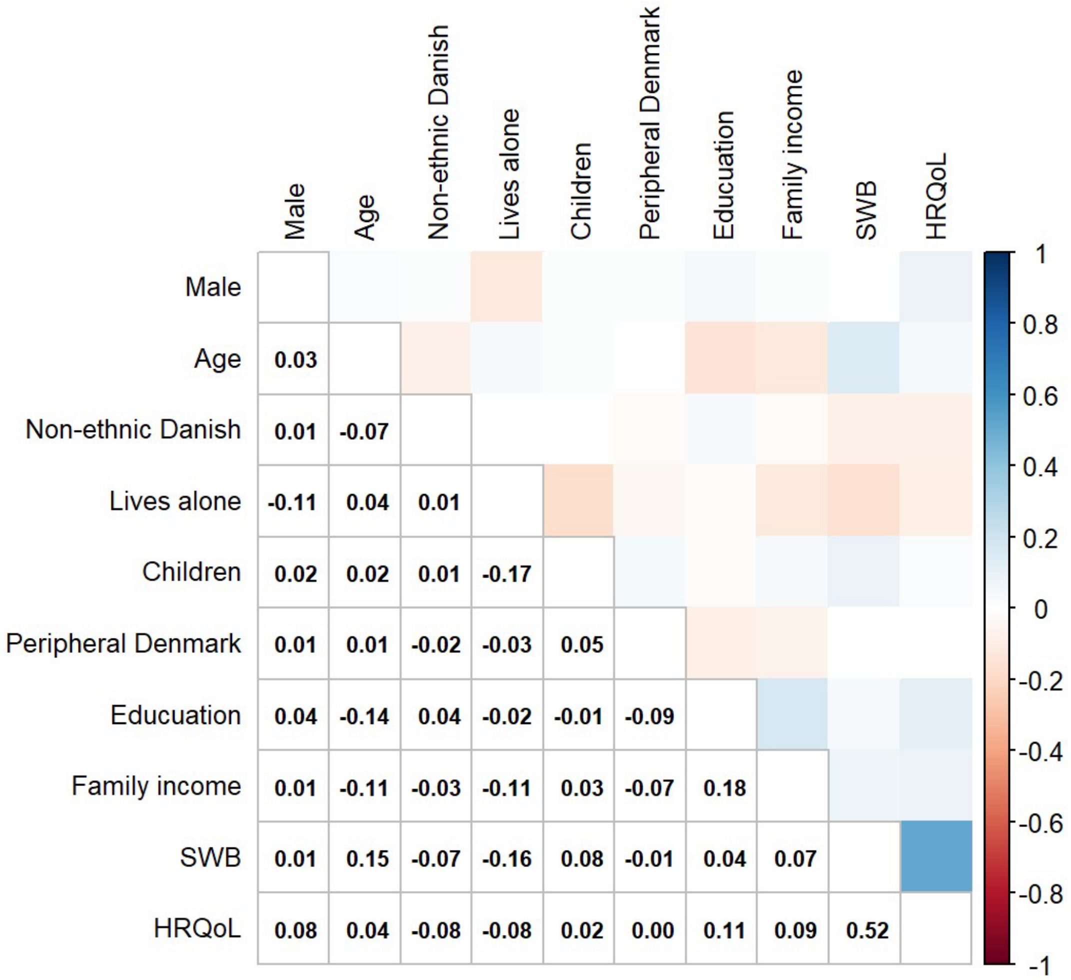

Fig. A4 Correlation plot

Note: The figure displays correlations between covariates. The upper half of the plot presents correlations by colours; the lower half presents the correlations in numbers.

References

1. EuroQol Research Foundation. EQ-5D-5L | Valuation | Crosswalk Index Value Calculator [Available from: https://euroqol.org/eq-5d-instruments/eq-5d-5l-about/valuation-standard-value-sets/crosswalk-index-value-calculator/
2. Jensen CE, Sørensen SS, Gudex C, Jensen MB, Pedersen KM, Ehlers LH. The Danish EQ-5D-5L Value Set: A Hybrid Model Using cTTO and DCE Data. Applied Health Economics and Health Policy. 2021:1-13.
3. Quan H, Li B, Couris CM, Fushimi K, Graham P, Hider P, et al. Updating and validating the charlson comorbidity index and score for risk adjustment in hospital discharge abstracts using data from 6 countries. American journal of epidemiology. 2011;173(6):676-82.
